# Three-Dimensional Near-Field Microwave Holography for Tissue Imaging

**DOI:** 10.1155/2012/291494

**Published:** 2012-04-09

**Authors:** Reza K. Amineh, Ali Khalatpour, Haohan Xu, Yona Baskharoun, Natalia K. Nikolova

**Affiliations:** Department of Electrical and Computer Engineering, McMaster University, Hamilton, ON, Canada L8S4K1

## Abstract

This paper reports the progress toward a fast and reliable microwave imaging setup for tissue imaging exploiting near-field holographic reconstruction. The setup consists of two wideband TEM horn antennas aligned along each other's boresight and performing a rectangular aperture raster scan. The tissue sensing is performed without coupling liquids. At each scanning position, wideband data is acquired. Then, novel holographic imaging algorithms are implemented to provide three-dimensional images of the inspected domain. In these new algorithms, the required incident field and Green's function are obtained from numerical simulations. They replace the plane (or spherical) wave assumption in the previous holographic methods and enable accurate near-field imaging results. Here, we prove that both the incident field and Green's function can be obtained from a single numerical simulation. This eliminates the need for optimization-based deblurring which was previously employed to remove the effect of realistic non-point-wise antennas.

## 1. Introduction

Microwaves have been used for imaging and detection ever since the technology to generate and receive them became available. Microwaves penetrate many objects and materials which are optically opaque such as living tissues, wood, ceramics, plastics, clothing, concrete, and soil. Various imaging methods have been developed in applications such as remote sensing, underground surveillance, concealed weapon detection, through-the-wall imaging, and nondestructive testing and evaluation. For a more complete coverage, the reader is referred to [1]. Many of the data-processing algorithms currently developed for near-field tissue sensing are rooted in these prior methods.

**Figure 1:**
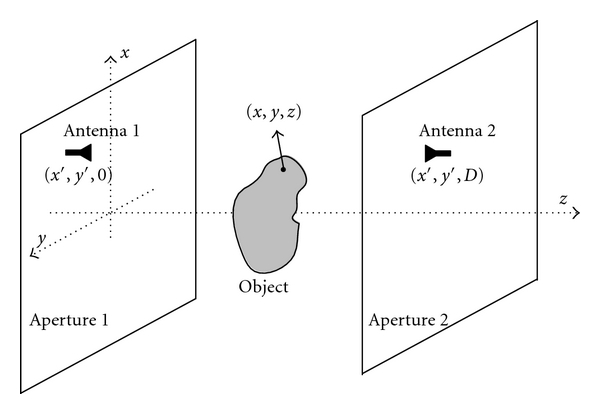
Microwave holography setup.

Microwave imaging of tissues dates back to the 1970s, when Larsen and Jacobi carried out extensive experiments with imaging canine kidneys [2]. They were successful in producing two-dimensional (2D) images where various tissues were clearly discernible. They measured the transmitted signal between two antennas facing each other along boresight (similar to the illustration in Figure 1). The imaged organ was scanned in a plane perpendicular to the line connecting the two antennas. Thus, the recorded signal was obtained as a function of two position coordinates, *x* and *y*, relative to a reference point on the imaged organ. Such data acquisition approach is often referred to as raster scanning. Raster scanning is also suitable for holographic reconstruction and is adopted in our work.

The major challenges in microwave tissue imaging include difficulties in coupling the microwave power into the tissue, significant tissue loss, relatively coarse resolution, significant tissue heterogeneity, and relatively low contrast between malignant and healthy tissues.

The active microwave systems currently considered for tissue imaging can be classified in four large groups [5]: optimization-based microwave imaging, confocal radar-based imaging, microwave tomography, and microwave holography.

The work here employs the latter approach. In modern microwave holography (e.g., see [6–9]), coherent (magnitude and phase) back-scattered signals are acquired on a surface, similarly to the conventional optical holography. However, the reconstruction of the object is based on a sequence of direct and inverse Fourier transforms (FTs). The data acquired on the surface is used simultaneously in a single reconstruction process to obtain the 3D reflectivity distribution of the object. It can be shown [10] that this reconstruction is based on the linear Born approximation. Microwave holography provides a framework where, with wideband frequency information, a 3D image of the object is obtained in quasi-real time. In the case of a single-frequency measurement, it provides a 2D image of the object's cross-section in a plane parallel to the acquisition plane (e.g., see [7]). Microwave holographic imaging based on rectangular and cylindrical aperture scanning has proven reliable and is employed in concealed weapon detection, for example, [6–9].

In [11], we extended the single-frequency 2D holographic image reconstruction to near-field microwave imaging. This method employs not only the back-scattered (as in [7]) but also the forward-scattered signals. The additional information from the forward-scattered signals improves the image quality and enables localization of the object in the range direction. In contrast to previous work, this method does not make any assumptions about the incident field such as plane, spherical, or cylindrical wave representations. The incident field is derived in a numeric form either through simulation or measurement. This is important in near-field imaging where the object is close to the antenna and the planar or spherical approximations of the illuminating wave are not valid. Green's function was still assumed to be a spherical wave.

In [10], we extend the 2D near-field holographic imaging technique in [11] to 3D imaging when wideband information is available. The proposed method has a number of distinct features and advantages compared to the previously proposed 3D holographic techniques. First, the method allows for incorporating forward-scattered signals in addition to the back-scattered signals. This additional information leads to more accurate reconstruction results and also allows for the significant suppression of image artifacts in the range direction. Second, the method allows for an incident-field distribution represented in numeric form. This distribution can be obtained either through simulation or through measurement with the particular antenna setup and medium. Third, it also allows for numeric input of Green's function, that is, the set of signals due to point scatterers in the given medium and received by the given antennas. These can be efficiently obtained through simulation as proposed here. The accurate representations of the incident field and Green's function for the particular problem are crucial in near-field imaging where analytical approximations such as plane or spherical waves are not adequate. Fourth, the numerical form of the incident field and Green's function necessitates a new inversion procedure. Previous 3D holography methods [7] relied on the analytical (exponential) form of the incident field and Green's function in order to cast the inversion expression in the form of a 3D inverse FT. This limits the technique to homogeneous background problems with far-zone measurements. Re-sampling of the data in *k*_*z*_ space is also necessary, which may lead to errors. This procedure is inapplicable with numeric representations of the incident field and Green's function. Instead, in [10] we solve a system of equations in each spatial frequency pair (*k*_*x*_, *k*_*y*_) and apply 2D inverse FT to the least-square solution at planes (slices) at all desired range locations. Note that the systems of equations have much smaller dimensions compared to the systems of equations in regular optimization-based microwave imaging techniques. This reduces the ill-posedness of the problem significantly. Thus, the 3D object is reconstructed as a set of 2D slice images in parallel planes along the range. The algorithm proves to be robust to high levels of noise.

In [10, 11], we employed copolarized dipole antennas to acquire the data. These antennas are small. Thus, the acquired data was assumed to be obtained from point sources/receivers. This allowed us to apply the holographic algorithms without any additional processing. However, this is not the case when in real practice we deal with non-point-wise antennas. In [12], we discussed that when we employ a real antenna structure to collect the data, additional processing is required prior to applying holographic image reconstruction. We employed blind de-convolution (deblurring) to eliminate the integration (blurring) effect of the non-point-wise antenna aperture. The major drawback of the deblurring processing is that it is based on nonlinear optimization procedures, which may fail to converge to the true solution.

Here, first we present a general vectorial formulation of 3D near-field microwave holography. Then, we show how the previously proposed scalar holographic algorithms can be derived from this more general formulation. Further, by using the reciprocity principle, we show that both the incident field and Green's function can be obtained in a single numerical simulation. This relatively simple theoretical development results in major progress in microwave near-field holography since not only we eliminate the required simulations to obtain Green's function, but also we do not need to apply “deblurring” signal processing. This is due to the fact that in this approach of obtaining Green's function, the antenna structure is part of the medium.

We examine the performance of the 2D and 3D image reconstruction techniques when employing TEM horn antennas [13] by a number of simulation and experimental examples.

## 2. Vectorial Holographic Microwave Imaging

The microwave holography setup considered here employs planar raster scanning. It consists of two antennas and an object in between as shown in Figure 1. When using the linear Born approximation [14], the scattered field is given by

(1) $$E^{\text{sc}}\left(r_{P}\right)\approx {∭}_{V_{Q}}^{}\underline{\underline{G}}\left(r_{P},r_{Q}\right)\cdot E^{\text{inc}}\left(r_{Q}\right)\left[k_{s}^{2}\left(r_{Q}\right)-k_{b}^{2}\right]dr_{Q},$$

where **E** ^sc^ is the scattered field, $\underline{\underline{G}}(r_{P},r_{Q})$ is Green's dyadic function, **E** ^inc^ is the incident field, *k*_*s*_ and *k*_*b*_ are the wavenumbers of the scatterer and the background media, respectively, and *V*_*Q*_ is the inspected volume. We assume that *k*_*b*_ is constant in *V*_*Q*_. The position vectors **r**_*P*_ and **r**_*Q*_ give the locations of the observation and scattering points, respectively.

### 2.1. The Forward Model

As shown in Figure 1, the antennas perform a 2D scan while moving together on two separate parallel planes positioned at *z* = 0 and *z* = *D*. Assume that at any measurement frequency *f*_*l*_ (*l* = 1,2,…, *N*_*f*_) we know the incident field **E** ^inc^(0,0, 0; *x*, *y*, *z*; *f*_*l*_) at any point **r**_*P*_ = (*x*, *y*, *z*) in the inspected volume when the transmitting antenna is at (0,0, 0). In addition, assume that all components of Green's tensor *G*_*i*_^*j*^(*x*, *y*, *z*; 0, 0, *D*; *f*_*l*_), *i*, *j* = *x*, *y*, *z*, are known for an *i*-polarized point source, at (*x*, *y*, *z*) and the *j*-polarized response at (0,0, *D*). For brevity, we introduce the notations:

(2) $$\begin{array}{c} E_{}^{\text{inc}}\left(x,y,z,f_{l}\right)\equiv E_{}^{\text{inc}}\left(0,0,0;x,y,z;f_{l}\right),\\ \underline{\underline{G}}\left(x,y,z,f_{l}\right)\equiv \underline{\underline{G}}\left(x,y,z;\;0,0,D;f_{l}\right). \end{array}$$

Let *E*_*j*_^sc^(*x*′, *y*′, *D*, *f*_*l*_),   *j* = *x*, *y*, *z*, be the *j*th component of the forward scattered *E*-field received at **r**_*Q*_ = (*x*′, *y*′, *D*). This implies that the transmitting antenna is at (*x*′, *y*′, 0) since it moves together with the receiving antenna. The back-scattered field is analyzed similarly with **r**_*Q*_ = (*x*′, *y*′, *D* = 0).

In a homogeneous or layered medium (where the layers are in *x*-*y* planes), the incident field and Green's tensor for the case where the antenna pair is at (*x*′, *y*′) can be obtained from those in (2) by a simple translation:

(3) $$\begin{array}{c} E_{}^{\text{inc}}\left(x^{\prime },y^{\prime },0;x,y,z;f_{l}\right)=E_{}^{\text{inc}}\left(x-x^{\prime },y-y^{\prime },z,f_{l}\right),\\ \underline{\underline{G}}\left(x^{\prime },y^{\prime },0;x,y,z;f_{l}\right)=\underline{\underline{G}}\left(x-x^{\prime },y-y^{\prime },z,f_{l}\right). \end{array}$$

Then, as per (1), each *j*-component (*j* = *x*, *y*, *z*) of the scattered field is written as

(4) $$\begin{array}{cc} & E_{j}^{sc}\left(x^{\prime },y^{\prime },D,f_{l}\right)\approx \\ & \;\overset{}{\underset{z}{\int }}\overset{}{\underset{y}{\int }}\overset{}{\underset{x}{\int }}f\left(x,y,z,f_{l}\right)\cdot \overset{}{\underset{m=x,y,z}{\sum }}g_{m}^{j}\left(x^{\prime }-x,y^{\prime }-y,z,f_{l}\right)dx\;dy\;dz, \end{array}$$

where

(5) $$\begin{array}{cc} & f\left(x,y,z,f_{l}\right)=k_{s}^{2}\left(x,y,z,f_{l}\right)-k_{b}^{2}\left(f_{l}\right),\\ & g_{m}^{j}\left(x,y,z,f_{l}\right)=E_{m}^{\text{inc}}\left(-x,-y,z,f_{l}\right)\\ & \;\;\;\;\;\;\times G_{m}^{j}\left(-x,-y,z,f_{l}\right)\;m=x,y,z. \end{array}$$

We refer to *f*(*x*, *y*, *z*, *f*_*l*_) as the contrast function. For simplicity, we assume that the contrast function is frequency-independent, that is, *f*(*x*, *y*, *z*) ≡ *f*(*x*, *y*, *z*, *f*_*l*_). The modification of the algorithm when dealing with dispersive media has been presented in [10]. Notice that (4) also implies that the scatterer is isotropic, that is, contrast function is independent of the polarization of the incident field.

In (4), the integral over *x* and *y* can be interpreted as a 2D convolution integral. Thus, the 2D FT of *E*_*j*_^sc^(*x*′, *y*′, *D*, *f*_*l*_)(*j* = *x*, *y*, *z*) is written as

(6) $$E_{j}^{\text{sc}}\left(k_{x},k_{y},D,f_{l}\right)\approx \overset{}{\underset{\text{z}}{\int }}F\left(k_{x},k_{y},z\right)\cdot \overset{}{\underset{m=x,y,z}{\sum }}G_{m}^{j}\left(k_{x},k_{y},z,f_{l}\right)dz,$$

where *F*(*k*_*x*_, *k*_*y*_, *z*) and *G*_*m*_^*j*^(*k*_*x*_, *k*_*y*_, *z*, *f*_*l*_) are the 2D FTs of *f*(*x*, *y*, *z*) and *g*_*m*_^*j*^(*x*, *y*, *z*, *f*_*l*_), *m* = *x*, *y*, *z*, respectively; *k*_*x*_ and *k*_*y*_ are the Fourier variables with respect to *x* and *y*, respectively.

To reconstruct the contrast function, we first approximate the integral in (6) by a discrete sum in *z* for the *N*_*z*_ reconstruction planes:

(7) $$\begin{array}{cc} & E_{j}^{\text{sc}}\left(k_{x},k_{y},D,f_{l}\right)\approx \overset{N_{z}}{\underset{n=1}{\sum }}F\left(k_{x},k_{y},z_{n}\right)\\ & \;\;\;\;\;\;\;\cdot \overset{}{\underset{m=x,y,z}{\sum }}G_{m}^{j}\left(k_{x},k_{y},z_{n},f_{l}\right)\Delta z\;j=x,\;y,\;z, \end{array}$$

where Δ*z* is the distance between two neighboring reconstruction planes.

### 2.2. Inversion Procedure

For the setup shown in Figure 1, there could be four antenna configurations when performing the raster scan: (1) antenna 1 and antenna 2 are both *x*-polarized (X–X case); (2) antenna 1 is *x*-polarized while antenna 2 is *y*-polarized (X–Y case); (3) antenna 1 is *y*-polarized while antenna 2 is *x*-polarized (Y–X case); (4) antenna 1 and antenna 2 are both *y*-polarized (Y–Y case).

Four complex *S*-parameters are acquired at the two antenna terminals at each frequency for each of the four polarization cases listed above. These four *S*-parameters constitute four separate scattered signals expressed in (1) (two reflection and two transmission coefficients). Thus, by performing wide-band measurements at *N*_*f*_ frequencies, from (7) *N*_*f*_ equations at each spatial-frequency pair (*k*_*x*_, *k*_*y*_) is obtained as

(8) $$\begin{array}{cc} & E_{j}^{\text{sc}}\left(k_{x},k_{y},D,f_{1}\right)\\ & \;\;\approx \overset{}{\underset{m=x,y,z}{\sum }}G_{m}^{j}\left(k_{x},k_{y},z_{1},f_{1}\right)F\left(k_{x},k_{y},z_{1}\right)\Delta z+\cdots \\ & \;\;\;+\overset{}{\underset{m=x,y,z}{\sum }}G_{m}^{j}\left(k_{x},k_{y},z_{N_{z}},f_{1}\right)F\left(k_{x},k_{y},z_{N_{z}}\right)\Delta z\\ & \;\;\;\;\;\;\;\;\;\vdots \\ & E_{j}^{\text{sc}}\left(k_{x},k_{y},D,f_{N_{f}}\right)\\ & \;\;\approx \overset{}{\underset{m=x,y,z}{\sum }}G_{m}^{j}\left(k_{x},k_{y},z_{1},f_{N_{f}}\right)F\left(k_{x},k_{y},z_{1}\right)\Delta z+\cdots \\ & \;\;\;+\overset{}{\underset{m=x,y,z}{\sum }}G_{m}^{j}\left(k_{x},k_{y},z_{N_{z}},f_{N_{f}}\right)F\left(k_{x},k_{y},z_{N_{z}}\right)\Delta z. \end{array}$$

Note that in (8), the subscript *j* denotes the polarization of the receiving antenna. For each configuration mentioned above, 4 × *N*_*f*_ equations are obtained. Then, the systems of equations for all four antenna configurations are combined to form a larger system of equations. In general, this results in a system of 12 × *N*_*f*_ decoupled equations, which must be solved for *F*(*k*_*x*_, *k*_*y*_, *z*_*n*_), *n* = 1,2,…, *N*_*z*_. Note that typically *N*_*z*_ < *N*_*f*_. 

At each spatial frequency pair (*k*_*x*_, *k*_*y*_), a system of equations as in (8) is solved in least-square sense to find *F*(*k*_*x*_, *k*_*y*_, *z*_*n*_), *n* = 1,2,…, *N*_*z*_. We emphasize that the system of equations obtained at each (*k*_*x*_, *k*_*y*_) is much smaller than the system of equations normally produced in optimization-based microwave imaging techniques. This significantly reduces the ill-posedness of our approach. Inverse 2D FT is applied to *F*(*k*_*x*_, *k*_*y*_, *z*_*n*_), to reconstruct a 2D slice of the function *f*(*x*, *y*, *z*_*n*_) at each *z* = *z*_*n*_ plane. Then, the normalized modulus of *f*(*x*, *y*, *z*_*n*_), |*f*(*x*, *y*, *z*_*n*_)|/*M*, where *M* is the maximum of |*f*(*x*, *y*, *z*_*n*_)| for all *z*_*n*_, is plotted versus *x* and *y* to obtain a 2D slice image of the object at each *z* = *z*_*n*_ plane, *n* = 1,2,…, *N*_*z*_. By putting together all *N*_*z*_ slice images, a 3D image of the object is obtained.

## 3. Obtaining Green's Dyadic Function

**Figure 2:**
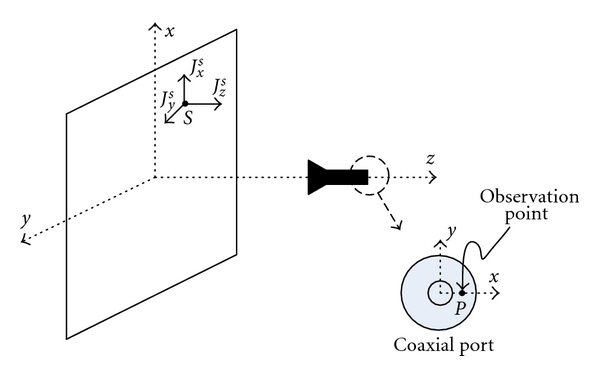
Illustration of the coaxial port for the receiving antenna with a point *P* on the port, on the *x*-axis and a point-scatterer at *S*.

Here, we assume that the antennas are fed by a coaxial cable. We also assume that the coaxial port is at an *x*-*y* plane, as illustrated in Figure 2, and the scattered field due to a point-scatterer at point *S* is sampled on the *x*-axis of the coaxial port at point *P*. Since the coaxial cable only supports TEM wave propagation along the cable (only the radial component of the *E*-field exists inside the cable), the sampled field at point *P* has an *x*-component only. Thus, the dyadic Green's function with the general expression of

(9) $$\underline{\underline{G}}\left(r_{P},r_{S}\right)=\left[\begin{array}{ccc} G_{x}^{x}\left(r_{P},r_{S}\right) & G_{y}^{x}\left(r_{P},r_{S}\right) & G_{z}^{x}\left(r_{P},r_{S}\right)\\ G_{x}^{y}\left(r_{P},r_{S}\right) & G_{y}^{y}\left(r_{P},r_{S}\right) & G_{z}^{y}\left(r_{P},r_{S}\right)\\ G_{x}^{z}\left(r_{P},r_{S}\right) & G_{y}^{z}\left(r_{P},r_{S}\right) & G_{z}^{z}\left(r_{P},r_{S}\right) \end{array}\right]$$

is simplified as

(10) $$\underline{\underline{G}}\left(r_{P},r_{S}\right)=\left[\begin{array}{ccc} G_{x}^{x}\left(r_{P},r_{S}\right) & G_{y}^{x}\left(r_{P},r_{S}\right) & G_{z}^{x}\left(r_{P},r_{S}\right)\\ 0 & 0 & 0\\ 0 & 0 & 0 \end{array}\right].$$

By convention, in the paired argument of Green's tensor, the 1st position vector denotes the observation point while the 2nd position vector denotes the excitation point.

To obtain this dyadic Green's function, one approach is to excite sequentially all *x*-, *y*-, and *z*-polarized sources at the positions *S*(*x*, *y*, *z*) of all the points (pixels) at all reconstruction planes and to obtain the *E*-field response resulting at point *P*. This approach is prohibitively inefficient. Instead, we employ the reciprocity principle. If a medium is reciprocal [14], the dyadic Green's function fulfills

(11) $$\underline{\underline{G}}\left(r_{P},r_{S}\right)={\underline{\underline{G}}}^{T}\left(r_{S},r_{P}\right),$$

where $\underline{\underline{G}}(r_{S},r_{P})$ implies that the source is at point *P* while the observation point is at point *S*. The superscript *T* in (11) denotes transposition. According to (11), we have

(12) $$\begin{array}{c} \begin{array}{c} \left[\begin{array}{ccc} G_{x}^{x}\left(r_{P},r_{S}\right) & G_{y}^{x}\left(r_{P},r_{S}\right) & G_{z}^{x}\left(r_{P},r_{S}\right)\\ 0 & 0 & 0\\ 0 & 0 & 0 \end{array}\right]\\ \;\;=\left[\begin{array}{ccc} G_{x}^{x}\left(r_{S},r_{P}\right) & G_{x}^{y}\left(r_{S},r_{P}\right) & G_{x}^{z}\left(r_{S},r_{P}\right)\\ G_{y}^{x}\left(r_{S},r_{P}\right) & G_{y}^{y}\left(r_{S},r_{P}\right) & G_{y}^{z}\left(r_{S},r_{P}\right)\\ G_{z}^{x}\left(r_{S},r_{P}\right) & G_{z}^{y}\left(r_{S},r_{P}\right) & G_{z}^{z}\left(r_{S},r_{P}\right) \end{array}\right]. \end{array} \end{array}$$

From (12), it follows that

(13) $$\begin{array}{c} G_{x}^{x}\left(r_{P},r_{S}\right)=G_{x}^{x}\left(r_{S},r_{P}\right),\\ G_{y}^{x}\left(r_{P},r_{S}\right)=G_{x}^{y}\left(r_{S},r_{P}\right),\\ G_{z}^{x}\left(r_{P},r_{S}\right)=G_{x}^{z}\left(r_{S},r_{P}\right),\\ G_{y}^{x}\left(r_{S},r_{P}\right)=G_{y}^{y}\left(r_{S},r_{P}\right)=G_{y}^{z}\left(r_{S},r_{P}\right)=0,\\ G_{z}^{x}\left(r_{S},r_{P}\right)=G_{z}^{y}\left(r_{S},r_{P}\right)=G_{z}^{z}\left(r_{S},r_{P}\right)=0. \end{array}$$

This indicates that we can excite the *x*-component at point *P* (coaxial port excitation) and observe the *x*-, *y*-, and *z*-components of the field at each point (pixel) at each reconstruction plane.

Notice that the incident field **E** ^inc^(*x*, *y*, *z*) is obtained in exactly the same way. Thus, only one simulation per polarization configuration is required to obtain both the incident field and the elements of Green's dyadic function. The final expression for the scattered field in terms of **E** ^inc^ only is

(14) $$\begin{array}{cc} & E_{}^{\text{sc}}\left(x^{\prime },y^{\prime },D,f_{l}\right)\\ & \;\;\approx \overset{}{\underset{\text{z}}{\int }}\overset{}{\underset{\text{y}}{\int }}\overset{}{\underset{\text{x}}{\int }}f\left(x,y,z,f_{l}\right)E_{}^{\text{inc}}\left(x^{\prime }-x,y^{\prime }-y,z,f_{l}\right)\\ & \;\;\;\;\;\cdot E_{}^{\text{inc}}\left(x^{\prime }-x,y^{\prime }-y,D-z,f_{l}\right)dx\;dy\;dz, \end{array}$$

where **E** ^sc^ is the forward scattered field due to the transmitting antenna at the plane *z* = 0 and captured by the receiving antenna at the plane *z* = *D*. Since we only consider the TEM mode inside the coaxial feed of the antenna, **E** ^sc^ is a scalar describing the radial component of that mode. In measurements, **E** ^sc^ is represented by the transmission scattering parameter of the two-port system formed by the two antennas and the imaged object. Note that (14) applies also to the case of a backscattered field if we set *D* = 0.

We emphasize that, in general, for each of the four possible mutual configurations of the two antennas (X-X, X-Y, Y-X, or Y-Y) described in Section 2.2, (14) provides a set of four equations for the four scattering parameters acquired with this two-port system.

## 4. Scalar Holographic Imaging [10, 11]

In the scalar holographic imaging, it is assumed that the antennas are linearly polarized, for example, *x*-polarized [10, 11]. In this case, the radiation field of the *x*-polarized antennas can be reasonably approximated by TM_*x*_ polarization. Thus, we consider the *x*-components of the incident and scattered *E*-fields only. This leads to a scalar Green's function which is the *G*_*x*_^*x*^ element of the full dyadic in (9). Thus, the expression in (4) (for nondispersive media as discussed in Section 2) is simplified as

(15) $$\begin{array}{cc} & E_{x}^{\text{sc}}\left(x^{\prime },y^{\prime },f_{l}\right)\\ & \;\approx \overset{}{\underset{z}{\int }}\overset{}{\underset{y}{\int }}\overset{}{\underset{x}{\int }}f\left(x,y,z\right)\cdot g_{0}\left(x^{\prime }-x,y^{\prime }-y,z,f_{l}\right)dx\;dy\;dz, \end{array}$$

where

(16) $$g_{0}\left(x,y,z,f_{l}\right)=E_{x}^{\text{inc}}\left(-x,-y,z,f_{l}\right)G_{x}^{x}\left(-x,-y,z,f_{l}\right).$$

Subsequently, the discretization, the construction of the systems of equations at each (*k*_*x*_, *k*_*y*_) pair employing all frequencies, and the reconstruction of the 2D images at all range locations are implemented in the same manner as explained above.

In order to perform 2D holographic imaging, it suffices to collect data at a single frequency [11]. The object is positioned at $z=\overset{̅}{z}$ and its thickness along the *z*-axis is assumed to be negligible. When using data at a single frequency, we can reconstruct a 2D image at the plane of the object. At a single frequency, (16) is simplified as

(17) $$E_{x}^{\text{sc}}\left(x^{\prime },y^{\prime },D\right)\approx \overset{}{\underset{y}{\int }}\overset{}{\underset{x}{\int }}f\left(x,y,\overset{̅}{z}\right)\cdot g_{0}\left(x^{\prime }-x,y^{\prime }-y,\overset{̅}{z}\right)dx\;dy.$$

Next, *E*_*x*_^sc^(*x*′, *y*′, *D*) is interpreted as a 2D convolution integral. This allows for the expression of the 2D FT *E*_*x*_^sc^(*k*_*x*_, *k*_*y*_, *D*) of *E*_*x*_^sc^(*x*′, *y*′, *D*) as

(18) $$E_{x}^{\text{sc}}\left(k_{x},k_{y},D\right)\approx F\left(k_{x},k_{y},\overset{̅}{z}\right)G_{0}\left(k_{x},k_{y},\overset{̅}{z}\right),$$

where $F(k_{x},k_{y},\overset{̅}{z})$ and $G_{0}(k_{x},k_{y},\overset{̅}{z})$ are the 2D FT of $f(x,y,\overset{̅}{z})$ and $g_{0}(x,y,\overset{̅}{z})$, respectively. Finally, the reconstructed reflectivity function of the object is obtained as

(19) $$f\left(x,y,\overset{̅}{z}\right)\approx F_{2\text{D}}^{-1}\left\{\frac{E_{x}^{\text{sc}}\left(k_{x},k_{y},D\right)}{G_{0}\left(k_{x},k_{y},\overset{̅}{z}\right)}\right\},$$

where *F*_2D_^−1^ denotes the inverse 2D FT. The reconstructed image can then be obtained as the magnitude of the reflectivity function $\left|f(x,y,\overset{̅}{z})\right|$.

Note that (19) is a formal reconstruction formula and it can be seen as the “maximum likelihood solution” if the collected data suffer from incompleteness and/or noise. The latter factors are often the cause for ill-posedness. However, the technique is very robust to noise and is not ill-posed. This is due to two factors. First, we provide sufficient sampling (in the Nyquist sense) in the scanned apertures. Second, we note that the incident field due to our antennas has a sharp peak in the middle of the reconstruction plane $z=\overset{̅}{z}$ while it quickly decreases toward the edges of this plane. This is mostly due to the dissipation of the power in the lossy background medium which causes significant attenuation for signals traveling along longer paths. Therefore, $G_{0}(k_{x},k_{y},\overset{̅}{z})$ varies smoothly as a function of *k*_*x*_ and *k*_*y*_ and the value of G_0_ is always significantly greater than zero for the considered wavenumbers. Hence, the division in (19) does not lead to ill-posedness of the inversion problem.

We should note that because of the finite size of the apertures, not all the wavenumbers (*k*_*x*_, *k*_*y*_) can be measured. This imposes lower and upper limits on *k*_*x*_ and *k*_*y*_, which in turn limits the cross-range resolution of the images as discussed in [11].

The collected data from each S-parameter can be processed using (19) to create an image of the object. In this case, we obtain four separate images with various resolutions. To reconstruct a single image, the datasets of all *S*-parameters can be used simultaneously where a least-square solution is sought as discussed in [11] and similarly to the inversion procedure given in Section 2.2.

## 5. Results

In this section, we present the results of 2D and 3D vector holographic imaging when employing real antennas. We obtain both the incident field and Green's function in a single numerical simulation as discussed in Section 3. Also, both tangential components of the field (*x*- and *y*-components) are considered on the reconstruction planes. Obviously, this leads to improved reconstruction results compared to the scalar holography in which only the co-polarized component of the field is considered on the reconstruction planes.

We employ simulation data to validate both 2D and 3D holography algorithms. However, due to the availability of only transmission *S*-parameters in our current measurement setup, we present experimental results only for 2D holography. We do not incorporate reflection *S*-parameters in the simulated 2D results for congruence with the measurements. According to our experience, the availability of the reflection *S*-parameters is crucial for 3D holography reconstruction. We note that the use of a multisensor array on the receiving side would enable 3D holography with transmission signals only; however, this system is currently under development.

### 5.1. TEM Horn Antenna Tailored for an Aperture Raster Scanning Setup

**Figure 3:**
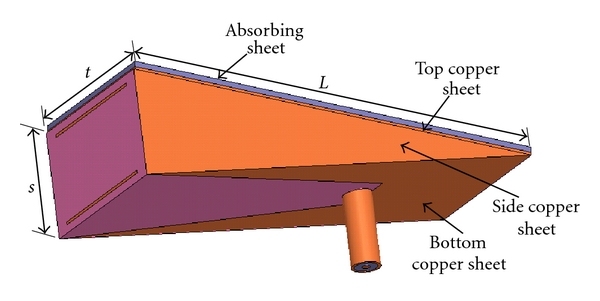
The TEM horn placed in a dielectric medium with relative permittivity of 10 with copper sheets on all outer surfaces except the front aperture and a microwave absorbing sheet on the top surface. *L* = 74 mm, *s* = 19 mm, and *t* = 30 mm.

The design, fabrication, and characterization of an ultra-wideband antenna for near-field microwave imaging of dielectric objects has been presented in [13] together with an imaging setup based on raster scanning. The focus here is on an application in microwave breast tumor detection. This TEM-horn antenna (shown in Figure 3) operates as a sensor with the following properties: (1) direct contact with the imaged body, (2) more than 90% of the microwave power is coupled directly into the tissue, (3) ultra-wideband impedance match, (4) excellent decoupling from the outside environment, (5) small size, and (6) simple fabrication.

The near-field imaging setup employs planar aperture raster scanning. It consists of two antennas aligned along each other's boresight and moving together to scan two parallel apertures. The imaged object lies between the two apertures.

### 5.2. 2D Holographic Imaging Results

To verify the proposed processing algorithms, we present simulation and experimental results first employing 2D holographic imaging. The properties of the background medium and the objects are chosen to be close to those of biological healthy and cancerous tissues, respectively.

**Figure 4:**
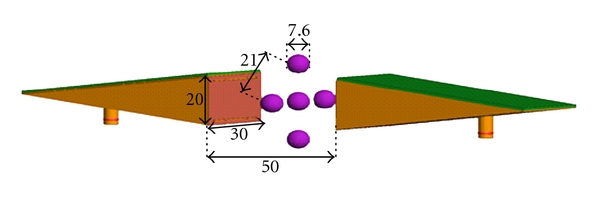
The simulation setup in FEKO. The dimensions are in mm.

In the first example, we use FEKO [15] simulation data from the 2D raster scanning of five spheres with *ε*_*r*_ = 15 and *σ* = 2 S/m embedded inside a medium with *ε*_*r*_ = 10 and *σ* = 1 S/m as shown in Figure 4. The diameter of the spheres is 7.6 mm and the center-to-center distance between the spheres on the periphery is 21 mm and the center-to-center distance between each sphere on the periphery and the central sphere is 15 mm. The distance between the antenna apertures is 50 mm.

The antennas perform 2D scan on a region of size 90 mm × 90 mm with spatial sampling rate of 5 mm. At each sampling position, the complex *S*_21_ is measured at 5 GHz, 7 GHz, and 9 GHz.

**Figure 5:**
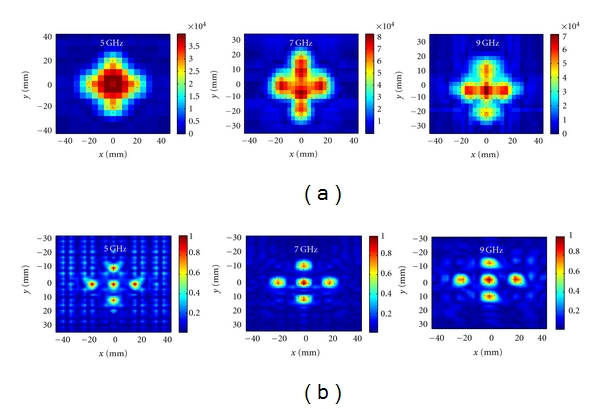
(a) Raw images obtained from calibrated |*S*_21_| at 5 GHz, 7 GHz, and 9 GHz, (b) normalized images after applying 2D holographic imaging at 5 GHz, 7 GHz, and 9 GHz.

Figure 5(a) shows the raw images created from calibrated |S_21_| obtained with the simulation setup in Figure 4. Calibration scheme has been discussed in [10, 11]. The spheres cannot be distinguished from each other at 5 GHz. At 7 GHz and 9 GHz, the resolution improves but still the image quality is poor. Then, we apply the holographic imaging algorithm to the complex-valued *S*_21_.  Figure 5(b) shows the reconstructed images. It is observed that the objects are vividly distinguished and the quality of the images is much improved.

**Figure 6:**
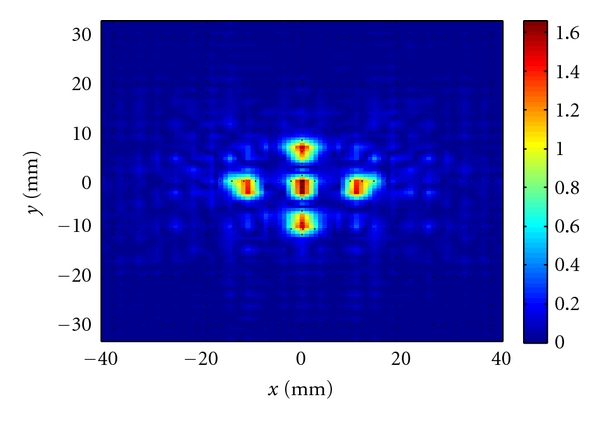
Image obtained when combining the data simulated at 5, 7, and 9 GHz in a single reconstruction process based on the least-square solution.

We also combined the data obtained from 5 GHz, 7 GHz, and 9 GHz in a single least-square-based image reconstruction algorithm as explained in Section 4.  Figure 6 shows the reconstructed image in which the objects are clearly discernible.

**Figure 7:**
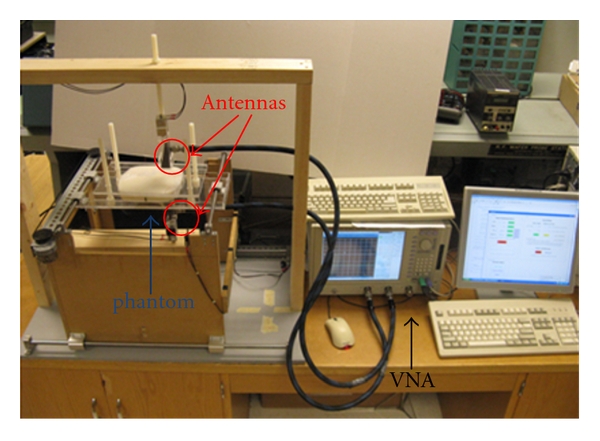
Experimental setup for aperture raster scanning.

In the second example, we use the measured data from the 2D raster scanning setup shown in Figure 7. Two objects are embedded in a brick-shaped phantom. The objects are two spheres with *ε*_*r*_ ≈ 50 and *σ* ≈ 4 S/m resembling tumors. They are made of alginate powder and are embedded inside the glycerin-based phantom emulating tissue with *ε*_*r*_ ≈ 7 and *σ* ≈ 1 S/m, at 7 GHz. The diameter of the spheres is about 10 mm and the center-to-center distance between the spheres is about 16 mm. The phantom is compressed between two thin plexi-glass sheets. The distance between the antenna apertures after this compression is 50 mm including the sheets. The antennas perform 2D scan on a region of size 70 mm × 70 mm with a spatial sampling rate of 5 mm. This scanning is performed by an automatic positioning system. At each sampling position, the complex transmission *S*-parameter between the two antennas (*S*_21_) is measured at 7 GHz and 9 GHz using a vector network analyzer (VNA). The VNA averaging and resolution bandwidth are set to 16 and 1 KHz, respectively. To improve the accuracy of the measurements (obtain signal well above the noise floor of VNA), an amplifier is employed in the transmitter side and a three-stage low-noise amplifier was employed in the receiver side.

**Figure 8:**
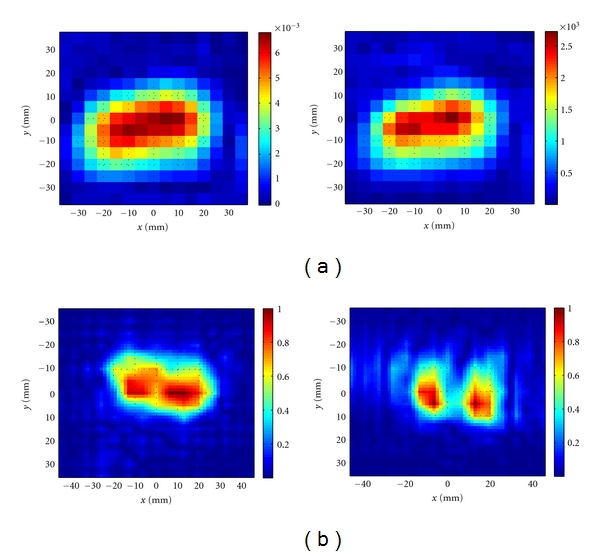
(a) Raw images obtained from calibrated |*S*_21_| at 7 GHz and 9 GHz, (b) normalized images after applying 2D holographic imaging at 7 GHz and 9 GHz.

Figure 8(a) shows the raw images created from the calibrated |S_21_| obtained from the measurement setup shown in Figure 7. The two tumor stimulants cannot be distinguished clearly. Then, we apply the 2D holographic imaging algorithm to the complex-valued *S*_21_.  Figure 8(b) shows the reconstructed images. As seen in this figure, the objects are clearly discernible.

**Figure 9:**
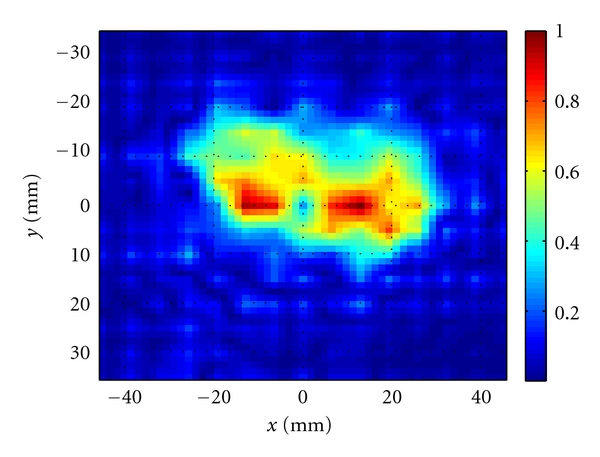
Normalized image obtained when combining the measured data obtained at 7 and 9 GHz in a single reconstruction process based on the least-square solution.

Also, similar to the simulation results, we employed the data obtained at 7 and 9 GHz in a single image reconstruction technique based on least-square solution.  Figure 9 shows the reconstructed image. The two objects can be distinguished well in this image.

### 5.3. 3D Holographic Imaging Results

**Figure 10:**
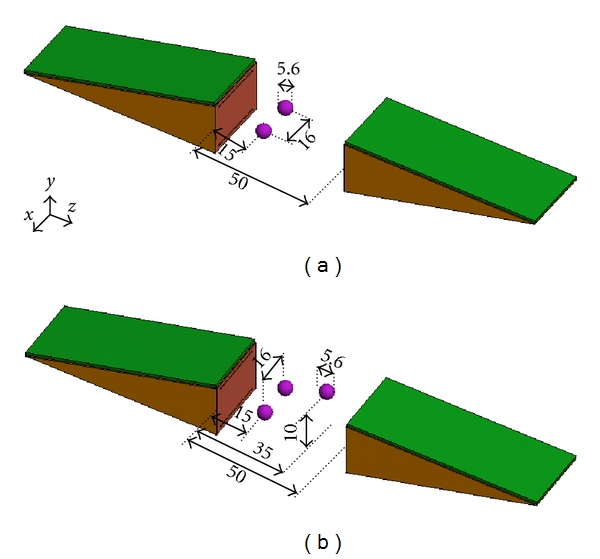
Simulation setup for two examples where spherical objects with diameter of 5.6 mm and properties *ε*_*r*_ = 15 and *σ* = 11 S/m are embedded inside a homogeneous background medium with properties *ε*_*r*_ = 10 and *σ* = 0.5 S/m; (a) two objects placed at the range position *z* = 15 mm with center-to-center distance of 16 mm and (b) two objects placed at the range position *z* = 15 mm with center-to-center distance of 16 mm and a third object centered at (0, 10, 35). The dimensions are in mm.

In this section, we present the simulation results for implementing 3D holographic image reconstruction for the two examples shown in Figure 10.

In the first example, as illustrated in Figure 10(a), two small objects with properties *ε*_*r*_ = 15 and *σ* = 1 S/m are placed at a range position of 15 mm inside a homogeneous background medium with *ε*_*r*_ = 10 and *σ* = 0.5 S/m. The diameter of the spheres is 5.6 mm and the center-to-center distance between them is 16 mm. Two antennas perform 2D raster scan on the rectangular apertures placed at *z* = 0 and *z* = 50 mm with size 72 mm × 102 mm. The spatial sampling rate in both *x* and *y* directions is 3 mm. The simulated reflection and transmission *S*-parameters are obtained from in the frequency range 3 GHz to 10 GHz with a step of 0.5 GHz. The incident field and Green's function for the antennas are obtained from a single simulation of one antenna in the absence of the objects as described in Section 3.

The 3D holographic image reconstruction algorithm is applied to the collected data. The contrast function is reconstructed on the planes the size of which is 140 mm × 140 mm. The planes are at *z* = 5,15,25,35, and 45 mm. The distance between the planes (10 mm) is larger than the minimum range resolution of 7 mm computed here from the 7 GHz bandwidth. As explained in [7, 10], the range resolution is inversely proportional to the bandwidth of the system. Although far-field propagation has been assumed in deriving the formula for range resolution, the expression gives a good approximation in the case of near-field measurements [10].

**Figure 11:**
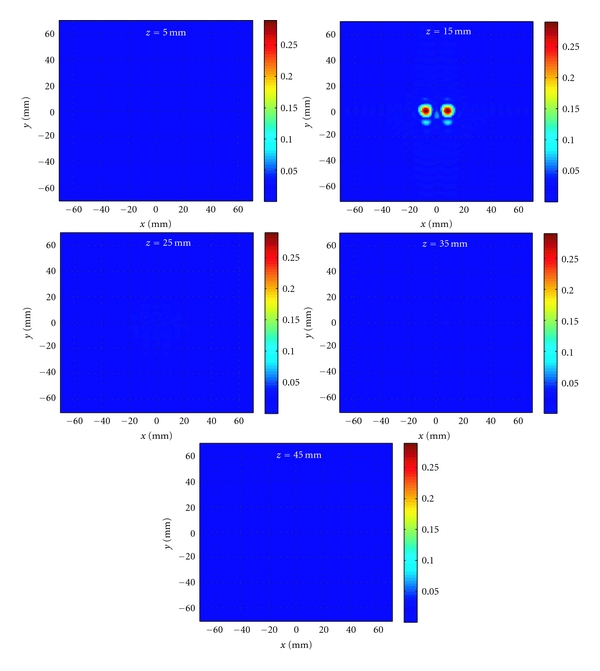
Reconstructed images for the example shown in Figure 10(a) after applying 3D holographic image reconstruction algorithm.

Figure 11 shows the reconstructed images. The two objects are reconstructed correctly in the plane at *z* = 15 mm. The image reconstructed at *z* = 25 mm shows weak artifacts and the images at other range positions correctly do not show the presence of any objects.

In the second example, as illustrated in Figure 10(b), three small spherical objects with properties *ε*_*r*_ = 15 and *σ* = 1 S/m are embedded between the two antennas inside a homogeneous background medium with *ε*_*r*_ = 10 and *σ* = 0.5 S/m. The diameter of the spheres is 5.6 mm. Two of the objects are placed at a range position of 15 mm and the center-to-center distance between them is 16 mm. The third object is centered at the position (0, 10, 35) mm. Two antennas perform 2D raster scan on the rectangular apertures placed at *z* = 0 and *z* = 50 mm. The size of the scanned apertures is 48 mm × 60 mm. The spatial sampling rate in both *x* and *y* directions is 3 mm. The simulated reflection and transmission *S*-parameters are obtained in the frequency range from 3 to 10 GHz with steps of 0.5 GHz. The incident field and Green's function for the antennas are obtained from a single simulation of one antenna in free space as described in Section 3.

The 3D holographic image of the contrast function is obtained on planes of size 140 mm × 140 mm at *z* = 5,15,25,35, and 45 mm.

**Figure 12:**
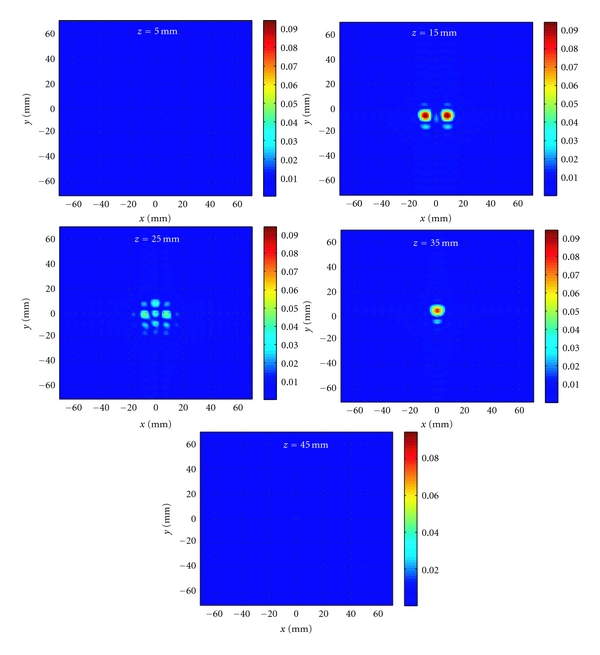
Reconstructed images for the example shown in Figure 10(b) after applying 3D holographic image reconstruction algorithm.

Figure 12 shows the reconstructed images. The three objects are reconstructed well at the correct positions *z* = 15 mm and *z* = 35 mm. The reconstructed image at *z* = 25 mm shows some artifacts. However, the maximum of the reconstructed contrast |*f*(*x*, *y*, *z*_3_)| (image at *z* = 25 mm) is more than two times smaller than the maximum of |*f*(*x*, *y*, *z*_2_)| reconstructed at *z* = 15 mm. The images at the other range positions correctly do not show the presence of any objects.

It is worth noting that the difference between the scales in the images of Figures 11 and 12 is due to the application of the artifact removal technique, first proposed in [10].

## 6. Conclusion

In this paper, for the first time, we presented the formulations for a full vectorial 3D microwave holography. We also discussed how this full vector formulation reduces to the scalar microwave holography when considering two linearly polarized antennas oriented in a copolarized manner. Furthermore, we described a new approach based on the reciprocity principle to obtain Green's function from the same simulation used to obtain the incident field. This significantly mitigates the computational burden to obtain Green's function as well as eliminates the need for implementing “deblurring” algorithms prior to microwave holography when employing non-point-wise antennas.

We employed our recently proposed TEM-horn antennas in a rectangular aperture raster scanning setup to acquire wideband *S*-parameters. The capabilities of the proposed processing techniques in reconstructing the shape of the objects in 2D and 3D spatial domains were demonstrated through a number of simulations and experiments. The algorithms can reconstruct the object's shapes in quasi-real time. The images obtained from both simulations and measurements suffer from some artifacts. The artifacts in the experimental results are larger. In general, they are due to the measurement noise caused by the environment and the equipment, by the slight mis-match between the simulated and experimental performance of the antennas, as well as finite apertures. The artifacts in the simulated data are weaker. They are due to the numerical noise in obtaining the *S*-parameters, incident field, and Green's function as well as the finite aperture sizes. Proper signal processing techniques can be employed to reduce the artifacts.

The presented techniques are promising in the microwave imaging of tissues where they can provide an initial guess for the interior of the tissue while further processing would be employed to take into account the heterogeneity of the tissues.
